# Validity of the SF-12 for Use in a Low-Income African American Community-Based Research Initiative (REACH 2010)

**Published:** 2008-03-15

**Authors:** Celia O Larson, David Schlundt, Kushal Patel, Margaret Hargreaves, Katina Beard

**Affiliations:** Department of Medicine, Meharry Medical College; Diabetes Research and Training Center, Vanderbilt University, Nashville, Tennessee; Department of Medicine, Meharry Medical College, Nashville, Tennessee; Department of Medicine, Meharry Medical College, Nashville, Tennessee; Nashville REACH 2010, Matthew Walker Comprehensive Health Center, Nashville, Tennessee

## Abstract

**Introduction:**

The objective of our study was to assess the psychometric properties of the Medical Outcomes Study's 12-Item Short Form Survey Instrument (SF-12) for use in a low-income African American community. The SF-12, a commonly used functional health status assessment, was developed based on responses of an ethnically homogeneous sample of whites. Our assessment addressed the appropriateness of the instrument for establishing baseline indicators for mental and physical health status as part of Nashville, Tennessee's, Racial and Ethnic Approaches to Community Health (REACH) 2010 initiative, a community-based participatory research study.

**Methods:**

A cross-sectional random residential sample of 1721 African Americans responded to a telephone survey that included the SF-12 survey items and other indicators of mental and physical health status. The SF-12 was assessed by examining item-level characteristics, estimates of scale reliability (internal consistency), and construct validity.

**Results:**

Construct validity assessed by the method of extreme groups determined that SF-12 summary scores varied for individuals who differed in self-reported medical conditions. Convergent and discriminate validity assessed by multitrait analysis yielded satisfactory coefficients. Concurrent validity was also shown to be satisfactory, assessed by correlating SF-12 summary scores with independent measures of physical and mental health status.

**Conclusion:**

The SF-12 appears to be a valid measure for assessing health status of low-income African Americans.

## Introduction

Clinicians and researchers frequently use the Medical Outcomes Study’s 12-Item Short-Form Survey Instrument (SF-12) to assess and monitor health-related quality of life. (The term "item(s)" henceforth will refer to SF-12 questions with the associated response categories.) The SF-12 measures eight attributes of functional health status: physical functioning, role limitations resulting from physical health problems, bodily pain, general health, vitality (energy and fatigue), social functioning, role limitations resulting from emotional problems, and mental health (psychological distress and psychological well-being). In addition, the SF-12 assesses overall physical and mental function using summary scales, Physical Component Summary Score (PCS-12) and Mental Component Summary Score (MCS-12), which are scored through comparison with population norms estimated from responses to the 1990 National Survey of Functional Health Status (1,2).

The SF-12 has been used to measure the health status of patients with a specific diagnosis (3-9) as well as that of the general population, including health plan enrollees and various age and ethnic groups (10-13). The survey has been administered by telephone (4,14), self-administered in a clinic setting (10,14,15), administered by personal interview in a clinic setting (8), and administered as a mail survey (14,16,17). There is growing interest and some evidence of validity for using the SF-12 as a measure of population health status for purposes of planning, implementing, and evaluating community health interventions (16). Although research has shown the SF-12 to be valid for specific demographic groups (10,11,14,18-20), few studies have examined the psychometric properties of the SF-12 when used with minority populations in the United States (15,17,21-22). The dearth of research regarding the use of the SF-12 for American minority populations underscores the importance of further research because it was developed and validated in an ethnically homogeneous, predominantly white population. As with any measure of health related to quality of life, it is important to understand the psychometric characteristics to assure that appropriate interpretation and adequate inferences are made about the population of interest (23).

In light of alarming disparities in the health of the disadvantaged, including minority groups and those with low incomes, community-based participatory research (CBPR) initiatives have become more prevalent as a means of improving population health, often replacing more conventional approaches. CBPR is a partnership approach that involves community members, researchers, and organization representatives in all stages of the research process, including identification of key issues, study design, development and implementation of interventions, evaluation, and dissemination of research findings. This approach is often developed and implemented through community coalitions and relies on decision-making processes that empower all partners to contribute their knowledge and expertise, thus facilitating a collaborative, equitable relationship with shared ownership and responsibilities. The basic principles of CBPR are community member engagement, using local knowledge in the design of interventions and investing community members in the process and products (24). Trust between researchers and community partners is critical, albeit a challenge in light of past research experience in which the community derived little or no benefit or feedback from study results (25). Research studies should employ research tools, surveys, and questionnaires that are sensitive to the culture and norms of the population, to its race/ethnicity, age, social class, language, reading level, or religious customs (25). Such research tools not only will ensure valid results but also will build confidence and trust in academic and community partnerships. In addition, if evidence of validity is demonstrated, measures such as the SF-12 or other health-related functional health indicators can be recognized as useful health status measurement tools to inform the effectiveness of community-based health interventions with specific racial/ethnic populations.

The purpose of our study was to assess the validity of the SF-12 for establishing a baseline for mental and physical health of African Americans within the context of a CPBR study, Nashville's Racial and Ethnic Approaches to Community Health (REACH) 2010 initiative, which seeks to reduce disparities in diabetes and cardiovascular disease between African Americans and whites (26). The analytic approaches presented here test the validity and reliability of the SF-12 instrument within the context of a larger community-based data collection effort.

## Methods

### Questionnaire

The SF-12 was implemented as a subset of 12 questions embedded within a 154-item survey (see Appendix for SF-12 questions). The 142 survey questions that accompanied the SF-12 measured several social and behavioral domains relevant to the individual's health status and health maintenance including health care use, exercise, nutritional habits, and self-care behaviors for those with diabetes. The 142 questions also included a subset of questions to test the validity of the SF-12, questions related to self-reported diseases and conditions (diabetes, obesity, and cardiovascular disease), self-perceived functional health (number of days in the past 30 days physical health or mental health were poor), and level of social support (1). The questions that accompanied the SF-12 also assessed demographic characteristics (age, sex, race/ethnicity, education, and income).

### Sample

The REACH 2010 population resides in 11 census tracts in the North Nashville area of Davidson County. The area comprises 13,081 predominantly African American households (i.e., 89% African American and 11% white), of which 30% are headed by single women (27). Twenty-five percent of African American residences in Davidson County are in North Nashville (27). On the basis of a 95% level of confidence and a confidence interval of 3%, we determined that 1087 responses would be needed to adequately represent the population in the geographic area. To assure adequate sample size for analysis of subgroups, such as age and sex, we determined we would need 392 respondents per group to detect a two-point difference at 80% power (alpha = .05) using a two-tailed test (28). We used the sampling procedure performed by Sampling Data Research Services, Inc, Atlanta, Georgia, which resulted in selection of all residential directory listings in the North Nashville area (N = 9000) to achieve the sample size needed to make multiple comparisons. The household eligibility criteria for respondent selection were 1) being aged 18 years or older, 2) living or staying in the sampled household, and 3) having a household in the targeted population area. Only one adult was interviewed per household using the “most recent birthday method” for respondent selection. This method has been shown to produce representative samples comparable to other more commonly used, but more complicated, methods, such as the Kish method (29). Respondents were excluded if they were too ill or cognitively impaired to complete the 30-minute survey or were non-English speaking.

### Methods

Trained interviewers conducted telephone surveys from June to September, 2001, on weekdays, from 4:00 PM to 8:00 PM. The interviewers received standardized training on the interview protocol as well as cultural sensitivity training. A computer-assisted telephone interviewing (CATI) system was used for data collection. CATI eliminates errors associated with other methods of gathering data and entering information into a database and assures randomization for initial calls and callbacks. Up to 10 calls were made to each number in an attempt to get one completed survey per household. A 10-call design was used to increase the likelihood of including younger and more mobile respondents, who are less likely to be at home and reached in a standard five-call design. If the interview was not conducted at the time of initial contact with the eligible respondent, it was rescheduled at a time convenient for the respondent. The process yielded a 34.9% adjusted response rate (N = 1721). The response rate was adjusted for the following: disconnected numbers (n = 2576), fax/modem numbers (n = 140), not a private residence (n = 443), respondent physically unable to answer the survey (n = 122), or respondent ineligible (n = 790). The refusal rate was 23.2% (n = 2087); however, the break-off rate (partially completed surveys) was only 2.6% (n = 237).

### Analysis Plan

To assess the adequacy of the SF-12 instrument for making inferences about the health status of our African American community sample, we performed item- and scale-level analyses. Item-level descriptive statistics were evaluated, including data completeness and floor and ceiling effects (i.e., the extent to which respondents score at the top or bottom of a scale). In addition, the scales were evaluated for internal consistency, reliability, floor and ceiling effects, and comparison with U.S. population norms.

Construct validity was assessed using the "extreme groups" technique and calculations of convergent and discriminant validity. The extreme groups method of construct validation determines the extent to which the scale scores correspond to another attribute of the sample in a meaningful way (30). For example, individuals who are obese should have lower physical function scores than individuals who are not obese. Individuals who have diabetes are more likely to have lower physical function than those who do not have diabetes. Likewise, individuals who are elderly should report lower physical-function health status than younger individuals. In regard to mental health status, it would be expected that those who have more social support would have a higher level of mental health functioning than those with no social support.

Convergent validity assesses the extent to which item scores correlate with their own hypothesized subscale scores, and discriminant validity assesses the extent to which item scores have a higher correlation with their hypothesized scales than with other scales in the questionnaire (30). Multitrait scaling analysis was performed to evaluate the item–scale correlations, corrected for item overlap with the scale (i.e., the correlation between each item and the total score was computed from the remaining items in that scale) (31) to prevent overinflated values. For example, an item that measures the extent to which emotional health affects daily activities should correlate more highly with the mental health summary scale than with the physical health summary scale.

Another useful assessment of validity when using an instrument in a specific population is concurrent validity, which is determined by assessing how well the scores correlate with other similar measures of the attribute. For example, SF-12 physical health summary scores and SF-12 mental health scores should correlate well with the number of days an individual perceived his or her physical health and mental health to be poor during a previous 30-day period.

## Results

A total of 1721 African American adults responded to our survey. Of these, 61% were female with an average age of 53.2 years (Table 1). Fifty-three percent reported at least a high school education, and 52.8% were employed. Comparison of this sample with U.S. Census 2000 demographics shows that the sample underrepresented younger adults (aged 18–24 years) and educated adults (post-high school education) and overrepresented older adults (aged ≥65 years).

As seen in Table 2, 11% of respondents reported having been previously diagnosed with diabetes, which is considerably higher than the U.S. average of 7% (32). In addition, based on self-reported height and weight, 31% of this sample was obese. Twenty-four percent reported their overall health as fair or poor (based on a five-point Likert scale [excellent, very good, good, fair, poor]). Thirty-five percent reported that they had had 1 day or more during the past month when their physical health was not good, whereas 23% reported that they had had 1 day or more during the past month when their mental health was not good. Our indicator of social support, measured by the number of friends or relatives who were available to offer emotional support when needed, showed that only 3% of the sample had no one available for help with emotional problems.

### Item-level characteristics

Data completeness was excellent for all SF-12 items, with less than 2% of respondents not responding to a question. Item distributions tended to be skewed, with more respondents scoring at a higher functional status. Five of the items showed notable ceiling effects, with more than 75% of respondents scoring the maximum possible score. These items showed very little disability among the sample in physical role functioning (two items), emotional role functioning (two items), and social functioning. No item showed floor effects (Table 3).

### Reliability and scale characteristics

The Physical Component Study (PCS12) and the Mental Component Study (MCS12) of the SF-12 demonstrated good internal consistency reliability, with alpha coefficients of .80 and .78, respectively (Table 3). In addition, there were no significant ceiling or floor effects for either scale. Ceiling effects were 3.20% for PCS12 and 14.17% for MCS12. Floor effects were 0.87% for PCS12 and 0.17% for MCS12.

### Convergent and discriminant validity

Multitrait analysis showed that the subscales of the SF-12 had good convergent and discriminant validity (31). Each item was entered so that a greater score indicated a more positive level of functioning. The MCS12 and PCS12 scales were formed by summing the items appropriate to each scale (33). As seen in Table 4, all items met the criterion for item-convergent validity (item–scale correlations ≥0.40 the standard established for the SF-36 questionnaire from which the SF-12 questionnaire was derived), and all item–scale correlations, adjusted for overlap, were higher with the item's own scale than with the other scale (i.e., the magnitude of the correlation is higher for each item with its hypothesized scale compared to the other scale) (33).

### Method of extreme groups

As shown in Table 5, one-way multiple analysis of variance revealed significant differences between age groups for PCS12 and MCS12. For PCS12, the two younger age groups (18–24 years and 25–44 years) did not significantly differ based on least-square difference (LSD) post hoc tests; however, each of these groups was found to be significantly different from the two older groups (45–64 and ≥65 years).

For the MCS12, LSD post hoc tests revealed that the oldest age group (65 years or older) had higher MCS12 scores compared with younger age groups (18–24 years, 25–44 years, 45–64 years). These younger age groups did not significantly differ on MCS12 scores.

Significant differences were also found for obesity for both PCS12 (*P* < .01) and MCS12 (*P* < .01) where those who were obese (BMI ≥30) reported a lower level of physical functioning and poorer mental health compared with those who were not obese.

Participants who reported being diagnosed with diabetes had a significantly lower level of functioning on the PCS12 compared with those who had not been diagnosed (*P* < .01). No significant differences were found for mental health functioning on the MCS12.

Social support was assessed by a single question: How many close friends or relatives would help you with your emotional problems or feelings if you needed it? Those who reported no support for their emotional problems reported significantly lower MCS12 scores than did those who reported having one or more friends or relatives (*P* < .01). We found no significant differences in presence or absence of social support for physical health functioning.

### Concurrent validity

Number of days during the past month that physical and mental health was poor correlated with PCS12 and MCS12 scores. As seen in Table 6, MCS12 correlated more substantially with the number of poor mental health days than with poor physical health days. Likewise, PCS12 correlated more substantially with number of poor physical health days than with mental health days.

### PCS12 and MCS12 sample comparisons with U.S. norms

The summary scores for our sample of African American adults ( Table 7) show that physical health scores were lower compared with U.S. scores for the general population. Mental health scores were found to be higher for this African American sample compared with U.S. scores. We found the standard deviations of the sample summary scores to be similar to the population norms.

## Discussion

Based on the results of this psychometric evaluation, SF-12 summary scores were reliable and valid for use with African Americans as measured within the context of a larger community-based study. At the item level, very good data completeness was obtained, demonstrating a willingness of this sample of adults to provide information about their health that bears well on the validity of the scale scores because loss of items from nonresponse bias is minimal. Ceiling effects were observed on several items, suggesting that these items may not be sufficiently sensitive to change for longitudinal use.

The two items that assessed the effects that physical health had on role functioning (Accomplished less than you would like [Q4]; Were limited in the kind of work or other activities [Q5]) and the two items that assessed the effects that emotional health had on role functioning (Accomplished less than you would like [Q6]; Didn't do work or other activities as carefully as usual [Q7]), as well as the social functioning item (How much of the time has physical or emotional problems interfered with your social activities [like visiting with friends or relatives] [Q12]), demonstrated that more than 75% of respondents had no impairment. As suggested by McHorney et al (34), it may be beneficial to replace the dichotomous response categories (i.e., presence or absence of limitations) with finer gradation of response categories. These results suggest that responses to these questions may not accurately represent the true level of role functioning of this sample. This may also be an artifact of the telephone mode of administration, in which respondents may minimize impairment of functioning in these areas when reporting verbally. Additionally, because the SF-12 was embedded in a longer questionnaire, the questions preceding the SF-12 items could have influenced SF12 responses to be more positive than if respondents were administered only the SF-12 survey. The instrument should be further tested at the item level among this population.

At the scale level, the MCS12 and PCS12 showed satisfactory reliability in internal consistency. In spite of the limitations at the item level, the scale scores did not show ceiling or floor effects that would support their use longitudinally. Construct validity was supported for the physical health summary score by differentiating between those individuals who would be expected to have a lower level of physical functioning based on being of older age, having diabetes, or being obese. Construct validity was also supported for the mental health summary score by differentiating between those who would be expected to have a lower level of mental health functioning based on the presence or absence of social support in their lives. Convergent and discriminant validity was evidenced by satisfactory intercorrelations between items and summary scale scores consistent with the construct. Items that pertained to emotional functioning, mental health, and social support were more highly correlated with MCS12 than with PCS12. Likewise, items that pertained to physical health, physical role functioning, general health, and bodily pain were more highly correlated with PCS12 than with MCS12. As might be expected, vitality was correlated fairly equally with both summary scores.

Concurrent validity was supported by the strong correlation of the MCS12 and PCS12 with the number of days respondents reported feeling in poor mental and physical health during the past month. These results show that the summary scores can be used to describe functional limitations in physical and mental health.

The physical health summary scores were marginally lower than the U.S. general population norms, consistent with the percentage of respondents who were older, had diabetes, or were obese. Interestingly, the mental health summary scores were found to be higher for this African American sample compared with the national norms of the U.S. general population. This may be an artifact of the item-level skewness observed for three of the five items that the MCS12 comprises, although ceiling effects were not observed at the scale level. It is possible that a telephone mode of administration elevated the MCS12 scores. Participants may have given more positive reports as a result of demand characteristics or social desirability when speaking with a telephone interviewer as opposed to completing an anonymous mail survey or speaking with a clinician. McHorney et al (35) determined that scores are higher when health status is reported by telephone compared with reporting health status by mail. Further research is needed to understand this potential effect.

We acknowledge several limitations of this study. The telephone methodology used to collect these data imposes limitations associated with noncoverage and nonresponse bias. A telephone survey methodology that employs a list of residential telephone subscribers has noncoverage bias against people without telephones, although the U.S. Census 2000 reports 94.5% telephone coverage in the geographic area we targeted (27). This methodology also biases against people who have unlisted telephone numbers and people who can be reached only by cell phone. Noncoverage bias could be reduced by combining a stratified residential telephone list method with a random-digit–dialing method that would include people who have unlisted numbers.

Nonresponse bias was evidenced by the low response rate (34.9%) and a high refusal rate (23%). This is consistent with declining response rates in national telephone surveys over the past several years (37). Also, households within highly urbanized areas of concentrated socioeconomic disadvantage are less likely to participate in telephone surveys (38).

Several additional factors may have contributed to the refusal rate within this study. The potential respondents were told the interview would take 30 to 40 minutes. This may have contributed to refusals among participants, particularly the young adult age group. Young adults (aged <25 years) were the most underrepresented in our study consistent with research that has shown that adults in this age group are among the hardest to reach in telephone surveys (36). Another contributing factor may have been that no monetary incentive or other compensation was offered for respondents' time to complete the survey. Finally, there may exist subgroups within our population of interest, such as Caribbean Islanders, Africans, and people of other origins, who may have been less likely to respond to telephone surveys as a result of language barriers or cultural influences.

A mail methodology or combination mail and telephone methodology may achieve higher response rates among low-income African Americans. Previous research has shown higher response rates to the SF-36 survey using a mail methodology than with a telephone methodology (35). Within a Medicaid population, mixed methodology surveys (user-friendly mailed surveys) with incentives (37), combined with telephone follow-up, have been shown to increase response rates (39). Incentives have been shown to enhance response rates among African Americans (40).

Further research could explore alternative and mixed methodologies within a low-income African American community. If administration of the SF-12 is embedded within a longer community survey, response rates may be less compromised by limiting the number of additional questions to keep the interview or mail survey as brief as possible. In addition, offering respondents incentives for survey completion may increase response rates regardless of mode of administration.

Collectively, results of our study demonstrate validity for cross-sectional use of SF-12 summary scales for measuring the health status of low-income African Americans. In addition, this study shows how simple procedures can be used to assess measurement properties of the survey instrument when used in a population that was not adequately represented in the original studies of the SF-12 instrument. Within a CPBR initiative, this approach will provide assurances to community members that will increase acceptance of the study results and will increase trust in academic–community partnerships by using or testing instruments that are culturally appropriate. It is imperative that future validity studies of new and existing instruments recruit African American participants as well as members of other racial and ethnic groups. We will focus our future work on the use of the SF-12 in longitudinal studies of health among low-income African Americans.

## Figures and Tables

**Table 1:** Demographic Characteristics, African American Respondents (N = 1721), Short-Form Survey (SF-12) and U.S. Census 2000 Population, North Nashville, Tennessee, 2001

**Characteristic**	Survey Respondents, %	**U.S. 2000 Census, %**
**Sex**		
Male	36.6	40.5
Female	61.0	59.5
Missing	2.4	NA
**Employment status**		
Employed	52.8	48.2
Unemployed	2.6	5.0
Not in work force	44.1	46.7
Homemaker	3.5	NA
Student	2.4	NA
Retired	30.3	NA
Unable to work	7.9	NA
Missing	0.5	NA
**Education**		
<High School	22.3	25.7
High school diploma or GED	30.7	34.2
Some college	24.1	25.2
4-year college degree or more	22.3	14.9
Data missing	0.6	NA
**Age, y (mean, 53.2; SD, 18.0)**		
18-24	6.9	23.2
25-44	30.4	32.8
45-64	33.9	25.7
≥65	28.8	18.3
Missing	0.0	—
**Annual income** a		
<10K	8.9	NA
≥10K to <20K	12.3	NA
≥20K to <25K	18.6	NA
≥25K to <35K	13.2	NA
≥35K to <50K	12.7	NA
≥50K	10.5	NA

GED indicates general equivalency diploma; NA, not available. Percentages may not total 100% because of rounding.

a Income figures not available from U.S. Census 2000.

**Table 2:** Self-Reported Health Status, African American Respondents (N = 1721), Short-Form Survey (SF-12), North Nashville, Tennessee, 2001

Health Status	Survey Respondents, %
**Has been told has diabetes**	
Yes	11.2
No	88.6
Data missing	0.2
**Obesity status, based on BMI**	
Obese (BMI >30)	31.4
Not obese (BMI ≤30)	60.9
Data missing	7.7
**Rates health status as**	
Excellent	10.3
Very good	26.9
Good	38.2
Fair/poor	24.0
Data missing	0.6
**Says physical health has not been good for the following number of days in the past 30 days**	
0 days	59.9
1-2 days	8.8
3-6 days	9.9
7-13 days	6.0
14-30 days	10.7
Data missing	4.6
**Says mental health has not been good for the following number of days in the past 30 days**	
0 days	72.8
1-2 days	5.2
3-6 days	6.9
7-13 days	3.5
14-30 days	7.0
Missing	4.6
**Has the following number of friends or relatives to help with emotional problems if needed**	
None	3.0
1	4.4
2	8.7
3 or more	79.8
Missing	4.1

BMI indicates body mass index.

**Table 3:** Results of 12-Item Short-Form Survey (SF-12), African American Respondents (N = 1721), North Nashville, Tennessee, 2001

**Item**	**Abbreviated Question^a^**	Missing Responses, %	Mean (SD)	Item Response Category Frequency^b^						Floor^c^, %	Ceiling^d^, %
				1	2	3	4	5	6		
**Q1**	Health rating in general	0.58	3.21 (0.99)	53	360	657	464	177	NA^e^	10.28	3.08
**Q2**	Limitations in moderate physical activities	1.16	2.59 (0.69)	199	318	1184			NA	68.8	11.56
**Q3**	Limitations in climbing several flights of stairs	0.93	2.46 (0.75)	267	389	1049			NA	60.95	15.51
**Q4**	Accomplished less because of physical health	1.57	1.77 (0.42)	396	1298				NA	75.42	23.01
**Q5**	Limited in work or activities because of physical health	1.22	1.78 (0.41)	384	1316				NA	76.47	22.31
**Q6**	Accomplished less as a result of emotional problems	1.22	1.83 (0.38)	292	1408				NA	81.81	16.97
**Q7**	Not careful in work or activities as a result of emotional problems	0.81	1.87 (0.34)	217	1490				NA	86.58	12.61
**Q8**	How much did pain interfere with work inside and outside home	0.81	4.31 (1.06)	41	143	116	354	1053	NA	61.19	2.38
**Q9**	How much of the time did you feel calm and peaceful	0.76	4.50 (1.34)	69	100	245	151	762	381	22.14	4.01
**Q10**	How much of the time did you have a lot of energy	0.41	4.15 (1.46)	107	181	274	226	652	274	15.92	6.22
**Q11**	How much of the time did you feel downhearted and blue	0.35	5.22 (1.23)	28	73	59	217	274	1064	61.82	1.63
**Q12**	How much of the time did physical health or emotional problems interfere with social activities	0.99	5.40 (1.25)	38	82	43	111	139	1292	75.07	2.21
**SF-12**	**Summary scores**	**α Coefficient**								**Floor^c^, %**	**Ceiling^d^, %**
**Physical Component Study**	Physical health summary score	0.80								0.87	3.2
**Mental Component Study**	Mental health summary score	0.78								0.17	14.17

a See Appendix for complete questions.

b Each response category in numerical order.

c Percentage of respondents at the lowest possible score.

d Percentage of respondents at the highest possible score.

e Not applicable because the question had only five possible responses.

**Table 4:** Results, Tests of Convergent and Discriminant Validity of Items (Questions) in the Physical Component Summary Scores (PCS12) and Mental Component Summary Scores (MCS12) on the 12-Item Short-Form Survey (SF-12), African American Survey Respondents (N = 1721), North Nashville, Tennessee, 2001

**Item**	**Abbreviated Question^a^**	**Correlation Coefficients**	
		**PCS12**	**MCS12**
**Q2**	Limitations in moderate physical activities	0.62^b^	0.30
**Q3**	Limitations in climbing several flights of stairs	0.59^b^	0.25
**Q4**	Accomplished less because of physical health	0.63^b^	0.35
**Q5**	Limited in work or activities because of physical health	0.65^b^	0.33
**Q8**	How much did pain interfere with work inside and outside home	0.57^b^	0.38
**Q1**	Health rating in general	0.49^b^	0.30
**Q10**	How much of the time did you have a lot of energy	0.52^b^	0.49
**Q6**	Accomplished less as a result of emotional problems	0.40	0.53^b^
**Q7**	Not careful in work or activities as a result of emotional problems	0.41	0.55^b^
**Q11**	How much of the time did you feel downhearted and blue	0.28	0.53^b^
**Q12**	How much of the time did physical health or emotional problems interfere with social activities	0.45	0.47^b^
**Q9**	How much of the time did you feel calm and peaceful	0.35	0.41^b^
**Summary**			
**Convergent validity**			
Range of correlations		0.49-0.65	0.41-0.55
Success rate, %		100^c^	100^c^
**Discriminant validity**			
Success rate, %		100^d^	100^d^

a See Appendix for complete questions.

b Item–scale correlation corrected for overlap (relevant item removed from its scale for correlation). These correlations also hypothesize being the highest in the same row.

c Percentage of item–scale correlations 0.40 or greater.

d Percentage of item–scale correlations (adjusted for overlap) higher within the item's own scale than in the other scale.

**Table 5:** One-Way Multiple Analysis of Variance by Group Differences in Age, Weight, Self-Reported Diabetes, and Social Support, African American Survey Respondents (N = 1721), North Nashville, Tennessee, 2001

Variable	PCS12 Mean^a^ (SD)	**F_*df *_(*P*)**	MCS12 Mean^a^ (SD)	**F_*df *_(*P*)**
**Age group, y^b^**				
18-24	51.93 (6.71)(a)	39.48_3,1565_ (<.01)	52.11 (8.63)(a)	8.15_3,1565_ (<.01)
25-44	50.65 (8.25)(a)		52.47 (9.54)(a)	
45-64	47.09 (10.32)(b)		53.40 (9.38)(a)	
≥65	44.35 (10.83)(c)		55.20 (8.50)(b)	
**Weight**				
Obese	46.39 (10.65)	18.86_1,1452_ (<.01)	53.00 (9.68)	5.23_1,1452_ (<.01)
Not obese	48.69 (9.42)		54.12 (8.76)	
**Has diabetes**				
Yes	42.04 (11.16)	79.54_1,1557 _(<.01)	53.41 (9.42)	0.12_1,1557_ (>.05)
No	48.47 (9.59)		53.64 (9.12)	
**Has social support**				
Yes	47.64 (10.04)	0.5512_1,1557_ (>.05)	53.77 (8.98)	14.28_1,1506_ (<.01)
No	46.77 (11.47)		48.79 (11.38)	

PCS12 indicates Physical Component Summary Score; MCS12, Mental Component Summary Score; F, F test.

a The Short Form-12 summary score norm ranges are 13-69 for the PCS12 and 10-70 for the MCS12.

b Significance of differences was determined by least-square difference post hoc tests. Mean values with different letters indicated in parentheses differ significantly at *P* <.01.

**Table 6:** Correlation of 12-Item Short-Form Survey (SF-12) Summary Scores With Other Mental and Physical Health Indicators, African American Survey Respondents (N = 1721), North Nashville, Tennessee, 2001

**Indicator, Physical or Mental Health**	**Correlation With No. Days Mental Health Not Good**	**Correlation With No. Days Physical Health Not Good**
Mental Component Study	−0.51^a^	−0.24^a^
Physical Component Study	−0.17^a^	−0.47^a^

a *P* <.001, based on Pearsons bivariate correlation coefficient.

**Table 7:** Comparison of 12-Item Short-Form (SF-12) Summary Scores for African American Survey Respondents (N=1721), with SF-12 U.S. Population Norms, North Nashville, Tennessee, 2001

**Survey Instrument**	**SF-12 Summary Scores, African American Survey Respondents (N = 1721)**		**SF-12 U.S. Population Norms**	

	Mean (SD)	Range	Mean (SD)	Range
Physical Component Study	47.58 (10.09)	13-66	50.12 (9.45)	13-69
Mental Component Study	53.60 (9.18)	16-72	50.04 (9.59)	10-70

## Appendix. SF-12 Interview Script, Health Survey of African American Adults, North Nashville, Tennessee, 2001

**Table:**

**Question No.**	**Question**
**Q1**	In general, would you say your health is excellent, very good, good, fair, or poor?
Now, I'm going to read a list of activities that you might do during a typical day. Please tell me if your health now limits you a lot, limits you a little, or does not limit you at all.	
**Q2**	Limitations in moderate activities, such as moving a table, pushing a vacuum cleaner, bowling, or playing golf?
**Q3**	Limitations in climbing several flights of stairs?
The following two questions ask you about your physical health and your daily activities.	
**Q4**	During the past 4 weeks, have you accomplished less than you would like as a result of your physical health?
**Q5**	During the past 4 weeks, were you limited in the kind of work or other regular daily activities you do as a result of your physical health?
The following two questions ask about your emotions and your daily activities.	
**Q6**	During the past 4 weeks, have you accomplished less than you would like as a result of any emotional problems, such as feeling depressed or anxious?
**Q7**	During the past 4 weeks, did you not do work or other regular activities as carefully as usual as a result of any emotional problems, such as feeling depressed or anxious?
**Q8**	During the past 4 weeks, how much did pain interfere with your normal work, including both work outside the home and housework? Did it interfere not at all, a little bit, moderately, quite a bit, or extremely?
The next questions are about how you feel and how things have been with you during the past 4 weeks. As I read each statement, please give me the one answer that comes closest to the way you have been feeling: is it all of the time, most of the time, a good bit of the time, some of the time, a little of the time, or none of the time?	
**Q9**	How much of the time during the past 4 weeks have you felt calm and peaceful?
**Q10**	How much of the time during the past 4 weeks did you have a lot of energy?
**Q11**	How much of the time during the past 4 weeks have you felt downhearted and blue?
**Q12**	How much of the time have your physical health or emotional problems interfered with your social activities, like visiting with friends or relatives?
